# Recurrent late-onset fibrotic capsular block syndrome after neodymium-yttrium-aluminum-garnet laser anterior capsulotomy: a case report

**DOI:** 10.1186/s12886-016-0261-x

**Published:** 2016-06-11

**Authors:** Joong Sik Koh, Young Bin Song, Won Ryang Wee, Young Keun Han

**Affiliations:** Department of Ophthalmology, Seoul National University College of Medicine, #101 Daehang-ro, Jongno-gu Seoul, 110-744 Korea; Department of Ophthalmology, Seoul Metropolitan Government-Seoul National University Boramae Medical Center, #20 Boramae-ro 5-gil, Dongjak-gu Seoul, 156-707 Korea

**Keywords:** Capsular bag distension, Capsular block syndrome, Cataract surgery, Neodymium-yttrium-aluminum-garnet laser posterior capsulotomy

## Abstract

**Background:**

Capsular block syndrome is an uncommon complication that occurs after cataract surgery. It is characterized by capsular distension, anterior intraocular lens displacement, anterior chamber shallowing, and unexpected myopic shifts. We report a case of recurrent fibrotic capsular block syndrome with Elschnig’s pearl-type posterior capsule opacification 10 months after neodymium-yttrium-aluminum-garnet (Nd:YAG) laser anterior capsulotomy.

**Case presentation:**

A 72-year-old Asian man complained of decreased visual acuity 5 years after undergoing phacoemulsification with posterior chamber lens implantation. Under slit-lamp examination, late postoperative capsular block syndrome was diagnosed and Nd:YAG laser anterior capsulotomy was performed. Ten months after anterior capsulotomy, the patient returned with decreased visual acuity and was diagnosed with recurrent fibrotic capsular block syndrome. Nd:YAG laser posterior capsulotomy was performed.

**Conclusions:**

We found that fibrotic capsular block syndrome could recur with Elschnig’s pearl-type posterior capsule opacification after Nd:YAG laser anterior capsulotomy for late postoperative capsular block syndrome without posterior capsule opacification.

## Background

Capsular block syndrome (CBS) is a rare complication that occurs after cataract surgery. It develops when the continuous curvilinear capsulorhexis (CCC) margin is occluded by the intraocular lens (IOL) optic [1]. This complication is characterized by distension of the capsular bag and accumulation of a liquefied substance inside the capsular bag [2]. Kim and Shin [2] classified CBS as either non-cellular, inflammatory, or fibrotic, according to clinical characteristics. Non-cellular CBS develops within a day after surgery and is caused by an ophthalmic viscosurgical device. Inflammatory CBS develops several days after surgery and is caused by an inflammatory reaction. Fibrotic CBS develops several months to years after surgery and is characterized by fibrosis over the entire circumference of the anterior capsule opening and posterior capsule opacification (PCO). Herein, we report a case of recurrent fibrotic CBS with Elschnig’s pearl-type PCO that occurred 10 months after neodymium-yttrium-aluminum-garnet (Nd:YAG) laser anterior capsulotomy, which is normally performed for inflammatory-like CBS without PCO or fibrosis.

## Case presentation

In July 2007, a 67-year-old man with an age-related cataract in his left eye underwent uneventful phacoemulsification with implantation of an Akreos Adapt posterior chamber intraocular lens (Bausch & Lomb, Rochester, NY, USA) in the capsular bag under Healon GV (Abbott Laboratories, Abbott Park, IL, USA). Phacoemulsification was performed through a temporal clear corneal incision, and there were no postoperative complications. One month after the operation, his uncorrected visual acuity (UCVA) was 20/30 with −0.5 D SPH = −1.25 D CYL × 60′. Three years after surgery, his UCVA was 20/20 with −0.75 D SPH = −1.00 D CYL × 70′. The anterior chamber was deep and the IOL was well-positioned within the capsular bag.

**Fig. 1:**
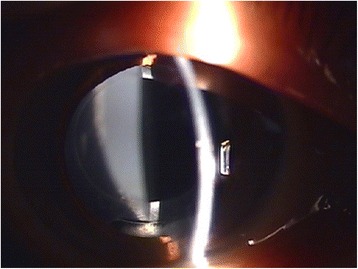
Slit lamp examination shows a milky-white substance accumulated behind the intraocular lens and posterior capsular bag distension

**Fig. 2:**
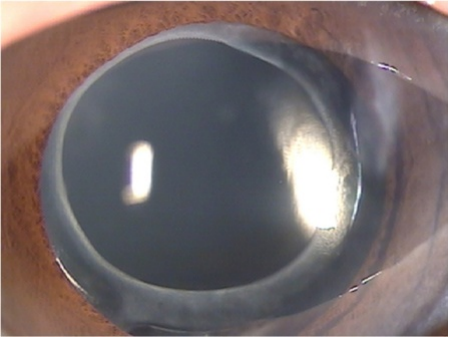
Slit lamp examination shows milky-white substance accumulation without capsular fibrosis at the first occurrence of capsular block syndrome

**Fig. 3:**
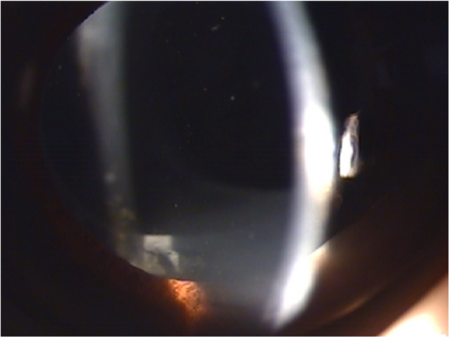
Six-o’clock Nd:YAG laser anterior capsulotomy was performed

**Fig. 4:**
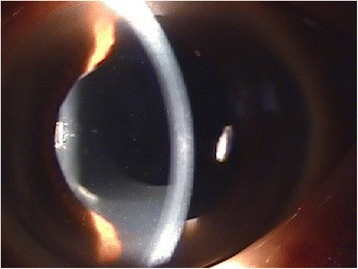
After Nd:YAG laser anterior capsulotomy, the milky-white fluid was emptied from the capsular bag into the anterior chamber, and the distended capsular bag was collapsed

In December 2011, the patient presented with decreased visual acuity of 20/100 with −2.75 D SPH = −2.25 D CYL × 70′, and his intraocular pressure (IOP) was 9.0 mmHg. Slit lamp biomicroscopic examination revealed a circumferential occlusion between the margin of the anterior capsule opening and the anterior surface of the peripheral IOL optic. There was a pool of turbid, milky-white fluid between the forward-displaced IOL and the posterior capsule. The posterior capsule was vaulted posteriorly, yet there was no PCO (Figs. 1 and 2). No cell or flare was observed in the anterior chamber, and no abnormalities were observed in the cornea, vitreous body, or retina. Capsular block syndrome was diagnosed, and posterior capsulotomy was scheduled to remove the substance. However, the Nd:YAG laser aiming beam could not be focused on the posterior capsule because of the intense opacity of the contents in the hyperdistended capsular bag. Alternatively, anterior capsulotomy was performed beyond the 6-o’clock edge of the IOL with an Nd:YAG laser (Fig. 3). A turbid fluid with minute white cells was emptied from the capsular bag anteriorly into the anterior chamber, and the distended capsule was restored (Fig. 4). One week after the procedure, the patient’s UCVA improved to 20/30 with −1.00 D SPH = −1.50 D CYL × 70′, and his IOP was 7.0 mmHg.

**Fig. 5:**
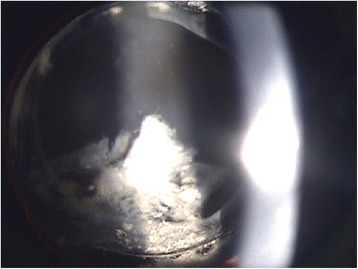
Slit lamp examination shows removed posterior capsule opacity and resolved capsular block syndrome following Nd:YAG laser posterior capsulotomy

Ten months after anterior capsulotomy, the patient returned with decreased visual acuity. His uncorrected visual acuity was 20/50 with −3.5 D SPH = −2.75 D CYL × 20′, and his IOP was 9.0 mmHg. Posterior capsular distension was observed with clear fluid in the bag. Elschnig’s pearl-type PCO and fibrosis along the entire capsule opening with clogging of the anterior capsulotomy opening were present. The patient was diagnosed with recurrent fibrotic capsular block syndrome, and Nd:YAG posterior capsulotomy was performed immediately. Posterior capsular opacity and fluid were emptied from the capsular bag posteriorly into the vitreous after the procedure, and the distended capsule was restored (Fig. 5). One week after the operation, his UCVA improved to 20/30 with −0.75 D SPH = −1.75 D CYL × 68′, and his IOP was 10.0 mmHg.

## Conclusions

Capsular block syndrome is an uncommon complication that occurs with posterior chamber, in-the-bag IOL implantation and CCC [3–5]. It was first described by Davison [3] as an early postoperative complication. Since then, other researchers have reported that CBS usually occurs during the early postoperative period; however, it can also occur during the late postoperative period [6–8]. Miyake et al. [9] classified CBS as intraoperative, early postoperative, and late postoperative. Late postoperative CBS may also be asymptomatic or undetected prior to development of PCO [10]. Late postoperative CBS occurs less frequently and has yet to be confirmed definitively.

According to the clinical characteristics described by Kim and Shin, postoperative CBS can be divided into three types: non-cellular (1 day after surgery; presents with a shallow anterior chamber and an anteriorly displaced IOL), inflammatory (several days after surgery; presents with distended capsular bag and inflammatory reaction), and fibrotic (late postoperative period; presents with circumferentially anterior capsule fibrosis and posterior capsule opacification) [2]. In our case, CBS with a turbid, milky-white fluid without capsular fibrosis was found in the first onset of CBS. It was neither non-cellular nor fibrotic; therefore, it was classified as a similar type of inflammatory CBS, despite no evidence of inflammation. Additionally, 10 months after anterior capsulotomy, there was fibrosis along the entire capsule opening and Elschnig’s pearl-type PCO. This led to the obvious conclusion of fibrotic CBS.

Clinically, CBS is characterized by capsular distension, anterior IOL displacement, anterior chamber shallowing, and unexpected myopic shifts. Reasons for myopic shift in CBS are multifactorial. Forward IOL displacement and possibly higher refractive indices of opalescent materials, compared with those of the IOL and vitreous humor, will move retinal images more anteriorly, thereby resulting in a myopic shift. Additionally, the enlarged space that fills with opalescent materials may act as either a phakic IOL or a piggyback IOL; this will also increase convergent power [11]. Other researchers have found that myopic shifts were not exhibited in late postoperative CBS [8, 12]. Bao et al. [7] showed that high resistance, induced by rigid fibrosis of the capsule, may prevent the IOL from being pushed forward and subsequent distension of the capsular bag. In our case, the myopic shift at the first onset of CBS can be explained by a forward-displaced IOL, increased convergent power due to opalescent materials, and no rigid fibrotic change. However, we also found myopic shifts in the recurrent fibrotic CBS with PCO. We think that this was caused by two different factors: (1) the previously distended anterior capsular may have had potential laxity to IOL anterior shifting and (2) Elschnig’s pearl-type PCO acting as a convex lens may have occurred during the myopic shift.

While some cases of early postoperative CBS can be resolved spontaneously within the first month, Nd:YAG anterior capsulotomy and posterior capsulotomy were suggested as treatment options for CBS [13]. Durak et al. [13] reported success rates for anterior capsulotomy (50 %, 5 of 10 eyes) and posterior capsulotomy (100 %, 5 of 5 eyes). According to this report, posterior capsulotomy, rather than anterior capsulotomy, is the recommended form of treatment; however, anterior capsulotomy is simpler, and there are times when posterior capsulotomy is not feasible owing to technical reasons [14].

In this case, we performed Nd:YAG laser anterior capsulotomy. It is difficult to focus on the posterior capsule while performing Nd:YAG laser posterior capsulotomy for late-onset CBS without PCO because of the opacity of the capsular bag contents and IOL vaulting. Furthermore, some people complain of floaters [15], and other complications, such as cystoid macular edema, severe inflammation, increased intraocular pressure or retinal detachment, have been reported after Nd:YAG laser posterior capsulotomy [16]. In some reports, Nd:YAG laser anterior capsulotomy was chosen as the primary treatment for late postoperative CBS [4, 13, 15]. We prefer anterior capsulotomy as a first line treatment for late-onset CBS without PCO. When PCO is present, we prefer to perform posterior capsulotomy. If posterior capsulotomy is not feasible owing to technical reasons, anterior capsulotomy followed by immediate laser posterior capsulotomy can be an alternative treatment.

The entrapped liquid in the capsular bag of patients with CBS has been biochemically analyzed. Suguira et al. [17] reported that sodium hyaluronate was the main component of the transparent liquid in the capsular bag in a case of early postoperative CBS. Namba et al. [18] detected a high protein concentration (primarily human serum albumin with small amounts of globulin fractions) in the milky-white fluid from two patients. Bao et al. [7] analyzed 6 years’ worth of passing milky-white liquid from patients with late postoperative CBS and reported that concentrations of Ca^2+^ and proteins proved to be crystals and crystalline peptides. These materials are thought to be elicited from residual lens epithelial cells (LECs), to increase osmotic pressure inside the capsular bag, and to cause greater liquid accumulation [5, 9]. However, it is unclear what factors lead to stimulation of residual LECs.

Although Kim and Shin [2] suggested that inflammatory CBS developed several days after surgery, our similar case of CBS without capsular fibrosis developed 4 years after surgery. This can explain how inflammatory CBS enables either late onset or slow progression. In this enclosed, stable condition, residual LECs are protected from metaplasia and proliferation. However, after anterior capsulotomy, we found fibrotic CBS with Elschnig’s pearl-type PCO. There was either LEC proliferation or anterior capsule fibrosis clogging the anterior capsulotomy opening; this led to a recurrence of fibrotic CBS. We found that if there were changes in lens capsular cavity, such as an interaction between aqueous humor and the capsular bag after anterior capsulotomy, LECs can be stimulated to metaplasia and proliferation. During our literature review, we found that Elschnig’s pearl-type PCO can occur when LECs are stimulated by the aqueous humor [19, 20]. For example, Elschnig’s pearl-type LECs have been known to proliferate from the adsorption of some extracellular matrix proteins in the aqueous humor [19, 20]. Despite this, we are unsure what kinds of substances were associated with our case. In our experience, intraoperative removal of LECs with complete cortex removal and capsule polishing is important in order to protect against CBS recurrence. Additionally, regular follow-up and re-evaluation of these patients are needed.

On the basis of this case, we found that fibrotic CBS and Elschnig’s pearl-type PCO could recur after Nd:YAG laser anterior capsulotomy for late postoperative CBS without PCO. To our knowledge, this is the first report of recurrent fibrotic CBS with Elschnig’s pearl-type PCO after Nd:YAG laser anterior capsulotomy.

## Abbreviations

CBS, capsular block syndrome; CCC, continuous curvilinear capsulorhexis; IOL, intraocular lens; IOP, intraocular pressure; LECs, lens epithelial cells; Nd:YAG, neodymium-yttrium-aluminum-garnet; PCO, posterior capsule opacification; UCVA, uncorrected visual acuity
